# Role of CD8+ cells in controlling replication of nonpathogenic Simian Immunodeficiency Virus SIVmac1A11

**DOI:** 10.1186/1743-422X-3-22

**Published:** 2006-04-03

**Authors:** Koen KA Van Rompay, Emily J Blackwood, Gary Landucci, Don Forthal, Marta L Marthas

**Affiliations:** 1 California National Primate Research Center, University of California, Davis, California, USA; 2 Division of Infectious Diseases, Department of Medicine, University of California, Irvine School of Medicine, Irvine, California, USA; 3 Department of Pathology, Microbiology and Immunology, School of Veterinary Medicine, University of California, Davis, California, USA

## Abstract

Infection of macaques with the avirulent molecular clone SIVmac1A11 results in transient low viremia and no disease. To investigate if this low viremia is solely due to intrinsic poor replication fitness or is mediated by efficient immune-mediated control, 5 macaques were inoculated intravenously with SIVmac1A11. Three animals that were depleted of CD8+ cells at the start of infection had more prolonged viremia with peak virus levels 1 to 2 logs higher than those of 2 animals that received a non-depleting control antibody. Thus, CD8+ cell-mediated immune responses play an important role in controlling SIVmac1A11 replication during acute viremia.

## 

Simian immunodeficiency virus (SIV) infection of macaques has proven useful for modeling HIV disease pathogenesis and intervention strategies [1-3]. While infection of macaques with most SIV isolates results eventually in an AIDS-like disease, there are also attenuated isolates and clones. SIVmac1A11 is a molecular clone originally derived from a virus isolate from an SIV-infected macaque that was also the source of virulent uncloned SIVmac251 isolates [4,5]. Although the kinetics are slower than for other isolates, SIVmac1A11 replicates well *in vitro *and is highly cytopathogenic (with induction of syncytia) in T-cell lines and rhesus macaque peripheral blood mononuclear cells (PBMC); SIVmac1A11 replicates well in macrophage cultures [6]. In early studies, it was observed that SIVmac1A11 inoculation of juvenile macaques resulted in transient viremia and no disease, even after prolonged follow-up for more than 12 years ([4]; unpublished observations). Subsequent studies documented that SIVmac1A11 inoculation of fetal and newborn macaques also resulted in transient viremia and no disease [7,8]. SIVmac1A11 has a tissue distribution distinct from that of virulent isolates [9].

Because of these unique properties, SIVmac1A11 has proven useful to study determinants of viral virulence. The genome of SIVmac1A11 has been sequenced, and recombination experiments revealed that differences in more than one region of the viral genome were responsible for the lack of virulence [5,10]. SIVmac1A11 has also shown promise as a live-attenuated vaccine in both infant and juvenile/adult macaques [10-13].

The transient low-level viremia (peak levels ≤ 4 to 5 log RNA copies per ml plasma) that results from SIVmac1A11 infection suggests either poor intrinsic replication fitness *in vivo *and/or relatively effective immune control. CD8+ cell depletion experiments (via administration of monoclonal antibody) have demonstrated the important role of CD8+ cell-mediated immune responses in controlling acute and chronic viremia with virulent SIV isolates (such as SIVmac251; [14]) and chronic viremia with the attenuated clone SIVmac239Δnef [15]; however, CD8+ cell depletion had no detectable effect on viremia in animals chronically infected with the more attenuated clone SIVmac239Δ3 [16] or with SIVmac1A11 (unpublished data). To our knowledge, no CD8+ cell depletion experiments have been performed during acute infection with nonpathogenic SIV isolates.

Accordingly, we sought to determine the role of CD8+ cell-mediated immune responses on acute SIVmac1A11 viremia. Animals in this study were juvenile rhesus macaques (Macaca mulatta; ~1 year of age), housed in accordance with American Association for Accreditation of Laboratory Animal Care Standards with strict adherence to the "Guide for the Care and Use of Laboratory Animals" [17]. When necessary, the animals were immobilized with ketamine HCL (Parke-Davis, Morris Plains, New Jersey) 10 mg/kg injected intramuscularly.

All 5 macaques were inoculated intravenously with a high dose of SIVmac1A11 (5 × 10^5 ^50% tissue culture infectious doses, grown in CEMx174 cells). Immediately before virus inoculation, 3 animals were depleted of CD8+ cells via administration of the anti-CD8 antibody cM-T807 at a dose of 50 mg/kg body weight (administered slowly intravenously); the same dose was repeated 3 weeks later. This dosage regimen, which is higher than the regimen used in previous CD8+ cell depletion studies [14,18], was selected because it gives more prolonged depletion of CD8+ cells (K. Reimann, personal communication). In the current study, CD8+ cells (both CD8+CD3+ T lymphocytes and CD8+CD3- NK cells) in peripheral blood were undetectable or low (< 1% of lymphocytes; ≤ 40 cells per μl blood) for 21 to 35 days after treatment (Fig. 1B,C). The remaining 2 animals received a control (i.e., non-depleting) human immunoglobulin preparation (Aventis Gammar-P I.V.) at the same dosage regimen (50 mg/kg at 0 and 3 weeks).

**Figure 1:**
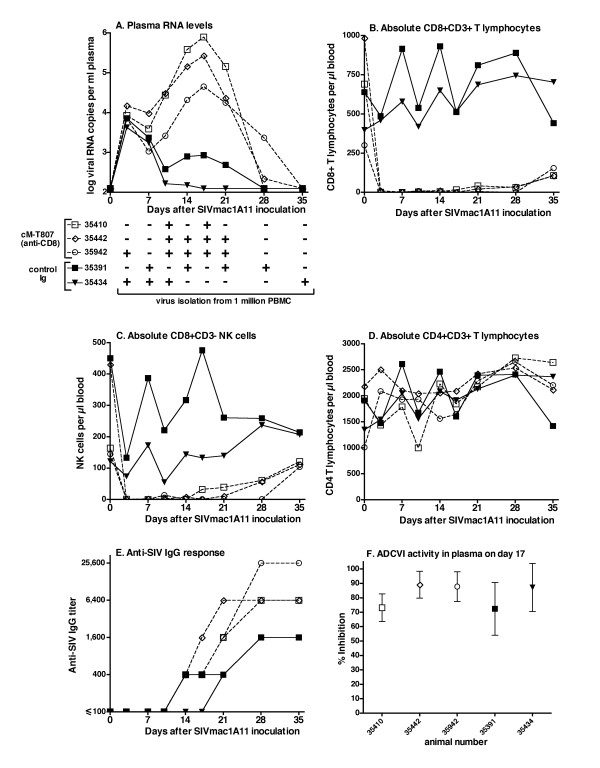
**Effect of CD8+ cell depletion on SIVmac1A11 infection: viral and immunologic parameters**. Five animals were inoculated with SIVmac1A11 at time zero. Three animals were CD8+ cell depleted via administration of cM-T807 while the other 2 animals received control antibody. (A) Viral RNA levels in plasma (measured by bDNA assay, with a limit of detection of 125 copies/ml; [18]). Results from virus isolation from 1 million PBMC, using CEMx174 cells and p27 measurement [34] are given as positive (+) or negative (-). The absolute counts of CD8+CD3+ T lymphocytes, CD8+CD3- NK cells and CD4+CD3+ T lymphocytes were measured according to flow cytometry techniques described previously [18], and are presented in graphs B through D, respectively. (E) SIV-specific IgG titers measured by a whole SIV ELISA [29]; although the CD8+ cell depleted animals made a faster response than the undepleted animals, from week 6 onwards, both animal groups had similar antiviral IgG titers (1: 6,400 to 1: 25,600). (F) Antiviral activity of plasma collected at 17 days after SIVmac1A11 inoculation as measured in a ADCVI assay, described in detail elsewhere (Forthal *et al*., submitted for publication). Briefly, CEMx174 cells were infected with SIVmac1A11 at a MOI of 0.01; 48 hours later, cells were plated in 96-well plates at 50,000 cells per well. Plasma samples (including negative and positive control samples) were added at a 1:100 dilution and human PBMC effector cells were added to obtain an effector:target cell ratio of 10:1. Five days later, SIV p27 was measured in supernatant fluid using a commercially available ELISA (Zeptometrix Corporation, Buffalo, NY). Percent inhibition by the plasma samples collected on day 17 was calculated relative to the level of virus replication in the presence of plasma collected on day zero (before SIVmac1A11 inoculation); the presented values represent mean +/- SEM of 4 separate assays (with effector PBMC of 4 different donors). In the absence of effector cells, no significant inhibition (≤ 11%) was observed (data not shown).

The 2 control-antibody treated animals had peak plasma viral RNA levels of 4 to 7 × 10^3 ^copies/ml at 3 days after virus inoculation (Fig. 1A). For one animal (number 35391), a second smaller peak of viremia was observed on day 17. The levels of viremia in these 2 control animals are thus similar to those described previously for SIVmac1A11-infected juvenile macaques [19]. The 3 CD8+ cell-depleted animals had viral RNA levels during the first 7 days that were indistinguishable from those of the control animals, suggesting that during these early stages, CD8+ cells had no detectable role in controlling SIVmac1A11 replication. However, after an initial decline, viral RNA levels in the CD8+ cell-depleted animals increased from day 10 onwards and reached peak levels of 45,000 to 790,000 on day 17; these values were 1–2 log higher than those of the control animals (p = 0.015, two-tailed t-test comparing day 17 values, and area-under-the curve values for day 0 to 35) and only ~1–2 log lower than peak viremia levels observed with the pathogenic molecular clone SIVmac239 [20-22]. Despite this higher viremia in the CD8+ cell-depleted animals, there were no significant changes in CD4+CD3+ T lymphocyte counts in peripheral blood (Fig. 1D); this study was not designed to monitor CD4+CD3+ T lymphocyte levels in gut-associated lymphoid tissue. Plasma viral RNA levels declined upon the return of CD8+ cells and became undetectable from 28 to 35 days of infection onwards throughout the rest of the observation period (> 6 months). These results indicate that CD8+ cells play a major role in controlling SIVmac1A11 replication because in their absence, peak viremia was higher and the acute viremia phase was significantly prolonged.

Because the cM-T807 antibody depletes both CD8+ T lymphocytes as well as NK cells, the relative contribution of each cell type could not be determined. CD8+ T cells and NK cells inhibit virus replication *in vitro *through a variety of mechanisms, including cytolytic and non-cytolytic pathways [23-25]. Most NK cells also have the low-affinity Fc-gamma III receptor (CD16), which triggers antibody-dependent cellular cytotoxicity (ADCC) and antibody-dependent cell-mediated virus inhibition (ADCVI). ADCVI is similar to ADCC but is a measure of virus inhibition, rather than target cell cytotoxicity. ADCVI has been observed *ex vivo *with serum and effector cells from HIV-infected humans and SIV-infected macaques [26](Forthal *et al*, manuscript submitted). Forthal *et al*. have also demonstrated that ADCVI-mediating antibodies can be found early during HIV-1 infection and reduce HIV-1 yield both by lysis of infected target cells and by the release of beta-chemokines from NK effector cells [27,28]. In the current study, the SIVmac1A11-infected animals had detectable antiviral IgG antibodies (as measured by whole SIV ELISA techniques; [29]) at ~2 weeks of infection, and the CD8+ cell-depleted animals had a more rapid increase in antibody titers, possibly due to more antigenic stimulation (Fig. 1E). Early plasma samples were also tested for ADCVI activity; pronounced inhibition (> 70%) was observed in plasma collected at 17 days of infection at a 1:100 dilution in all animals (Fig. 1F). Thus, some of the loss of viremia control following CD8+ cell depletion could be due to the loss of CD8+ NK cells that would likely serve as ADCVI effector cells.

Levels of interleukin-12 and interferon-α were measured in plasma using commercial ELISA-kits (monkey IL-12 ELISA, U-CyTech, Utrecht, the Netherlands; human interferon-α ELISA kit, PBL Biomedical Laboratories, Piscataway, NJ). Although variable levels were detected for both cytokines, there was no correlation with virus levels (data not shown).

In conclusion, this experiment demonstrated that the acute low-level viremia of SIVmac1A11 which is observed following inoculation of untreated animals cannot be explained solely by poor intrinsic replication fitness of the virus; instead, immune responses that are dependent on CD8+ cells limit the magnitude and duration of acute viremia. Viremia of SIVmac1A11 has always been observed to be transient (~2–6 weeks), even following inoculation of fetal and newborn macaques [7,8]. This indicates that these antiviral immune responses are not abrogated or prevented from emerging during acute SIVmac1A11 viremia and suggests that there is relatively little or no virus-induced immunosuppression. Rather, the anti-SIVmac1A11 immune responses appear able to induce a long-term asymptomatic infection [10]. This is in contrast to infection with virulent SIV isolates, for which irreversible damage to the immune system appears to occur early during the course of infection [9,30-32]. Accordingly, further experiments that combine avirulent strains such as SIVmac1A11 infection with selective depletions of immune cell populations may prove to be a useful and sensitive model to further unravel precisely the immune responses that are important to control viremia, but that may be difficult to detect during infection with virulent isolates. Attempts to boost or preserve such immune responses may lead to immunotherapeutic strategies that are more effective in achieving long-term control on viremia of virulent virus isolates, including HIV-1.

## Competing interests

The author(s) declare that they have no competing interest.

## Authors' contributions

KVR designed and coordinated the study, and drafted the manuscript; EB performed and analyzed viral and immunological assays; GL and DF performed and analyzed the ADCVI assays; MM participated in the design and interpretation of the study. All authors helped with and approved the final manuscript.
